# Acute Hemorrhagic Rectal Ulcer Presenting With Severe Lower Gastrointestinal Bleeding: A Case Report of an Emerging Entity

**DOI:** 10.7759/cureus.73465

**Published:** 2024-11-11

**Authors:** Batoul Abdallah, Ahmad Moussawi, Don C Rockey, Kassem Barada

**Affiliations:** 1 Internal Medicine, American University of Beirut, Beirut, LBN; 2 Gastroenterology and Hepatology, American University of Beirut Medical Center, Beirut, LBN; 3 Gastroenterology and Hepatology, Medical University of South Carolina, Charleston, USA; 4 Gastroenterology and Hepatology, American University of Beirut, Beirut, LBN

**Keywords:** colonoscopy, hematochezia, hemoclip, lower gastrointestinal bleeding, rectal ulcer

## Abstract

Acute hemorrhagic rectal ulcer (AHRU) is a rare but potentially life-threatening condition. We present the case of a 74-year-old man who developed sudden massive hematochezia and hypotension during hospitalization for fever of unknown origin. He was known to have alcohol-related liver cirrhosis, hypoalbuminemia and coronary artery disease (CAD) and was on daily aspirin. He was transfused and transferred to intensive care. Esophagogastroduodenoscopy (EGD) revealed no abnormalities, while colonoscopy showed two deep round ulcers in the distal rectum, one of which was spurting blood, promptly and successfully managed with hemoclip placement. There was no recent history of shock, constipation, or nonsteroidal anti-inflammatory drug (NSAID) use. A diagnosis of AHRU was made. The patient had no rebleeding but died two weeks later of septic shock. Gastroenterologists should consider AHRU in elderly patients with risk factors for AHRU such as prolonged bed rest, CAD, hypoalbuminemia, renal failure and anti-thrombotic drug use, who develop in-hospital lower gastrointestinal bleeding. Suggestive endoscopic findings are solitary or multiple rectal ulcer(s), with circumferential, round, Dieulafoy-like or geographical appearance and normal surrounding mucosa and location within 10 cm from the dentate line. Other etiologies should be excluded, and endoscopic hemostasis is often successful. It is important to recognize this entity and diagnose it early to decrease its associated morbidity and mortality.

## Introduction

Acute hemorrhagic rectal ulcer (AHRU) has been reported as the most common cause of inpatient onset lower gastrointestinal bleeding, according to a recent cohort study from Japan [1]. It typically affects hospitalized elderly patients with multiple comorbidities and presents with sudden, painless, severe hematochezia. Diagnosis is made through colonoscopy, which often shows one or more rectal ulcers with various morphologic appearances. AHRU has been reported in the East but is often overlooked in Western countries with only a few reported cases, which might delay appropriate diagnosis and treatment. Thus, increasing awareness of this syndrome is essential to ensure timely and effective management of severe lower gastrointestinal (GI) bleeding in critically ill patients.

## Case presentation

**Figure 1:**
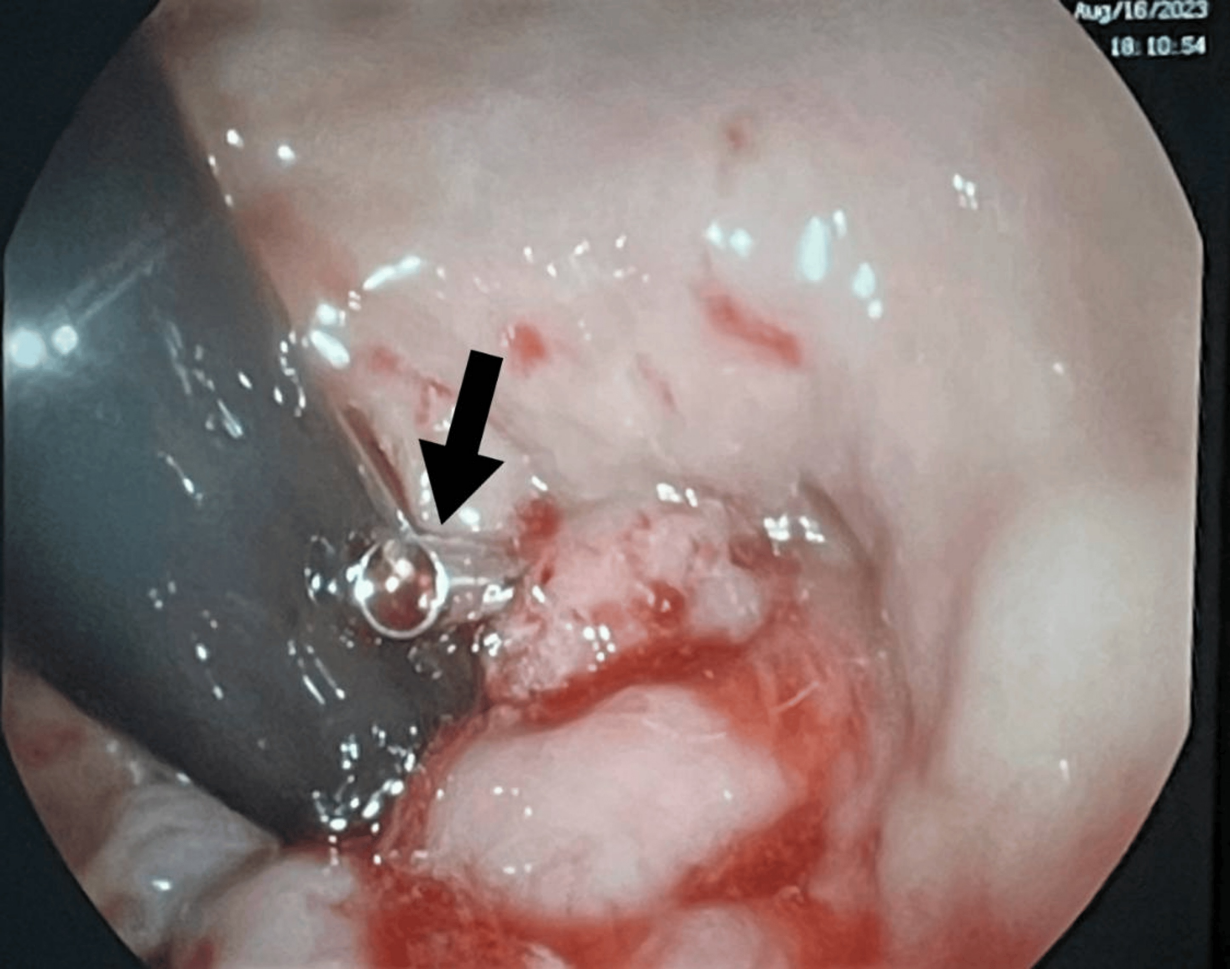
Hemoclip placement over the spurting vessel seen in the rectal ulcer bed with excellent hemostasis. Arrow indicates hemoclip.

A 74-year-old man developed sudden severe hematochezia and hypotension while being hospitalized for a workup of fever of unknown origin (FUO). The patient had had fever for two weeks prior to admission. Upon bleeding, his blood pressure and pulse changed from 106/71 mmHg to 70/41 mmHg and 80 to 115 bpm, respectively. He had an initial hemoglobin value of 11 g/dL which dropped to 8 g/dL after bleeding. His platelet count was 31,900/μL, creatinine was 0.86 mg/dL, international normalized ratio (INR) was 2.4, and his partial thromboplastin time (PTT) was 38.7 seconds. Physical exam including abdominal exam was unremarkable except for digital rectal exam (DRE) which showed fresh blood but no enlarged hemorrhoids or anal fissure. He was known to have alcohol-associated liver cirrhosis with hypoalbuminemia (25 g/L) and coronary artery disease, and he was on daily aspirin. He was transfused three units of packed red blood cells (PRBCs) and one unit of platelets and transferred to the intensive care unit. Esophagogastroduodenoscopy revealed no abnormalities, and colonoscopy showed two deep round ulcers in the distal rectum, just proximal to the dentate line (Video 1). One ulcer was spurting blood and was promptly managed with a hemoclip placement over the spurting vessel with excellent hemostasis (Figure 1). Biopsies were not obtained from the ulcers during colonoscopy.

**Video 1:** Retroflexion in the rectum showing two deep round ulcers just proximal to the dentate line. One ulcer was actively spurting blood. Black arrow indicates bleeding ulcer. White arrow indicates nonbleeding ulcer.

The patient did not have recent shock, constipation, history of pelvic radiotherapy, nonsteroidal anti-inflammatory drug (NSAID) use, or rectal trauma. A colonoscopy performed for anemia workup one week prior to presentation at another hospital was negative. A diagnosis of acute hemorrhagic rectal ulcer (AHRU) was made. Gastrointestinal bleeding stopped after endoscopic intervention; however, the patient passed away two weeks later from septic shock.

## Discussion

AHRU was initially reported in the East, particularly in Japan and Taiwan, where it may be the most common cause of in-hospital onset lower gastrointestinal bleeding [1,2]. However, recent reports indicate an increasing recognition of AHRU in the USA and the West [2]. AHRU might have been underreported in the West as making a diagnosis may be challenging. It typically presents as an acute rectal ulceration with minimal changes to the surrounding mucosa and is found close to the anal verge [3]. Thus, it can be easily missed during colonoscopy [3].

The differential diagnosis of bleeding rectal ulcers includes solitary rectal ulcer syndrome (SRUS), stercoral ulcers, infectious rectal ulcers, ischemic proctitis, trauma-induced rectal ulcers, proctopathy related to drugs or radiation and AHRU. Obtaining a careful history of trauma, NSAIDs use, constipation and radiotherapy along with the colonoscopy findings helps in differentiating AHRU from other entities.

Colonoscopy findings of AHRU may include solitary or multiple rectal ulcer(s), with circumferential, round, Dieulafoy-like or geographical appearance with normal surrounding mucosa, usually located within 10 cm from the dentate line [3]. Histopathologic examination of AHRU shows epithelial denudation, necrosis, hemorrhage and thrombi in the epithelial and stromal vessels [3]. These findings are similar to those observed in patients who had gastrointestinal tract necrosis secondary to cardiovascular dysfunction [4]. Like upper gastrointestinal tract stress ulcers, AHRU may occur after a stress-induced disruption to the blood circulation within the rectum [4].

Successful endoscopic hemostasis of AHRU may be achieved using electrocautery, argon plasma coagulation, local injection of pure ethanol or epinephrine, hemoclips, suture ligation, or gauze tamponade [5]. By contrast, angiographic embolization and emergency surgery are associated with high rates of mortality and rebleeding [6].

Rebleeding from AHRU is common, with rates ranging from 24.2% to 59.4% [7,8], typically happening six to nine days after initial hemostasis [9]. According to different studies, risk factors for rebleeding include coagulation abnormalities, severe comorbid conditions, a Charlson Comorbidity Index (CCI) of 4 or higher, hypoalbuminemia and whole circumferential ulcers [5,8,10,11]. Additionally, a recent study found that a history of blood transfusion prior to the first endoscopic examination increases the risk of rebleeding [9].

Inpatients with AHRU-related bleeding have a higher mortality rate than those with non-AHRU-related bleeding. In a study of 72 patients with AHRU, the in-hospital mortality rate was greater in the AHRU group compared to the non-AHRU group (18.0% vs. 8.3%, p = 0.02), with hypoalbuminemia identified as the only significant risk factor for in-hospital mortality [12]. Additionally, another study involving 36 patients with AHRU and ICU admission found that thrombocytopenia was a risk factor for mortality at four weeks [13].

Our patient had several risk factors for developing AHRU, including prolonged hospitalization and bed rest, ischemic heart disease, hypoalbuminemia and the use of aspirin. He was successfully treated with an endoscopic intervention and did not experience rebleeding; however, he died two weeks later from septic shock, reflecting a poor outcome. He had two specific risk factors for a poor prognosis: hypoalbuminemia and thrombocytopenia.

AHRU should be considered in hospitalized elderly patients with prolonged bed rest and those with cerebrovascular disease, ischemic heart disease, hypoalbuminemia, renal failure and anti-thrombotic drug use who develop in-hospital acute profuse lower GI bleeding [1,14]. Diagnosis hinges on clinical features, risk factors and characteristic findings on endoscopic examination along with exclusion of alternative etiologies [3].

## Conclusions

AHRU typically occurs in critically ill hospitalized elderly patients and typically presents with acute, severe, painless hematochezia. A high level of suspicion and a thorough retroflexion in the rectum during colonoscopy are crucial for identifying those ulcers and allowing timely intervention. Endoscopic hemostasis remains the most successful treatment option. Despite successful endoscopic hemostasis, the risk of rebleeding is high and the outcome may be unfavorable.
